# Benzene at 200: from a simple ring to a universe of fused aromatic carbon

**DOI:** 10.1039/d6sc90122k

**Published:** 2026-06-18

**Authors:** Nazario Martín, Ben L. Feringa

**Affiliations:** a Departamento de Química Orgánica I, Facultad de Química, Universidad Complutense Avda. Complutense s/n 28040 Madrid Spain nazmar@ucm.es; b IMDEA-Nanociencia Calle de Faraday, 9 28049 Madrid Spain; c Stratingh Institute for Chemistry, University of Groningen 9747 AG Groningen The Netherlands b.l.feringa@rug.nl

## Abstract

Nazario Martín and Ben L. Feringa introduce the cross-journal themed collection celebrating 200 years since the discovery of benzene.
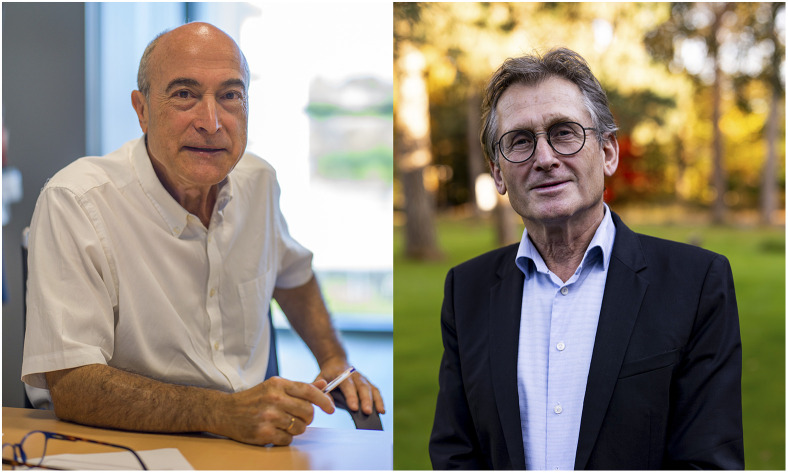

In 1825, Michael Faraday reported the isolation of a new hydrocarbon—then termed “bicarburet of hydrogen”—from the residues of illuminating gas.^1^ Communicated to the Royal Society of London, this discovery marked the beginning of a conceptual and molecular revolution. What appeared to be a simple, colourless liquid would ultimately redefine the foundations of chemistry and enable the emergence of modern materials science.

Benzene’s early behaviour defied classification. Its remarkable stability, despite a high degree of unsaturation, challenged prevailing ideas of chemical bonding. The resolution of this paradox came with the cyclic structural proposal of August Kekulé,^2^ which established the foundations of structural theory and introduced a new way of thinking about molecules—not as static assemblies, but as systems defined by connectivity and symmetry. From this insight emerged the concept of aromaticity, now central to chemistry.

The development of quantum mechanics provided a deeper understanding of this phenomenon. The work of Linus Pauling^3^ and others formalised the notion of electron delocalisation, transforming benzene into a paradigm for chemical bonding. Aromaticity became more than a structural motif: it became a guiding principle linking stability, reactivity, and electronic structure across molecular and extended conjugated systems.

Two centuries after its discovery, benzene is best understood not as an isolated molecule but both as the archetype and at the origin of a vast family of π-conjugated architectures. This themed collection, spanning journals of the Royal Society of Chemistry, situates benzene within this broader continuum, focusing on systems constructed through the fusion of its fundamental unit: the hexagonal aromatic ring.

The first level of this structural expansion is found in polycyclic aromatic hydrocarbons (PAHs), whose synthesis and conceptual development were shaped by pioneers such as Richard Scholl and Erich Clar.^4^ In these systems, the interplay between local aromatic units and global π-delocalisation gives rise to properties that depend sensitively on size, topology, and edge structure. PAHs thus represent a bridge between discrete molecules and extended carbon frameworks and have played a central role in the development of organic electronics.

This bridge has evolved into the field of nanographenes, where atomically precise control enables the design of carbon-based materials with tailored properties. By extending and shaping fused benzene frameworks, chemists can modulate electronic gaps, induce chirality like in the helicenes, and engineer reactivity with molecular precision. These advances exemplify a broader shift in chemistry—from synthesis as construction to synthesis as design—where function emerges from controlled architecture.

Beyond finite systems, the formal fusion of benzene rings leads to new forms of carbon allotropy. Fullerenes and carbon nanotubes, discovered in the late twentieth century, revealed that aromatic carbon could adopt curved and tubular geometries while preserving electronic delocalisation.^5^ These discoveries expanded the dimensionality of carbon chemistry and demonstrated that the principles derived from benzene apply far beyond planar molecules.

Graphene represents the limiting case of this progression: a two-dimensional, atomically thin lattice composed entirely of fused benzene rings. Isolated by Andre Geim and Konstantin Novoselov,^6^ it embodies the ultimate extension of aromaticity into a continuous material. Its exceptional mechanical, electronic, and optical properties have redefined the landscape of materials science and inaugurated the broader field of two-dimensional systems.

Taken together, PAHs, nanographenes, fullerenes, nanotubes, and graphene form a coherent hierarchy of structures spanning molecular to macroscopic scales and zero-to two-dimensional systems. All are rooted in the same fundamental principle: the stabilising and structuring power of delocalised π-electrons within a hexagonal carbon framework. Benzene thus serves not only as a molecule, but as a unifying concept across chemistry and materials science.

This conceptual reach is matched by benzene’s practical impact. It remains a central platform in the synthesis of polymers, pharmaceuticals, dyes, and functional materials, underpinning technologies that define modern life. At the same time, its classification as a Group 1 carcinogen by the International Agency for Research on Cancer^7^ highlights the dual nature of chemical progress, where utility and risk must be carefully balanced.

The contributions in this themed collection reflect the continuing evolution of benzene chemistry. Rather than providing a comprehensive survey, they illustrate how the principles derived from benzene continue to guide contemporary research—from fundamental questions of (anti)aromaticity to advances in on-surface synthesis and high-resolution characterisation techniques that enable direct visualisation of molecular structures. These developments bring an unprecedented level of precision to the study of aromatic systems, closing the gap between theoretical constructs and experimental reality.

Looking forward, the descendants of benzene are uniquely positioned to address major scientific challenges. Their structural tunability and electronic versatility make them central to emerging technologies in energy, nanoelectronics, and biomedicine. Yet, beyond these applications, benzene retains a deeper significance.

For generations of chemists, benzene has been an entry point into the language of molecular science—a first encounter with symmetry, delocalisation, and the interplay between structure and function. Its simplicity is deceptive: within its six-membered ring lies a conceptual richness that continues to inspire curiosity and creativity.

Two hundred years after its discovery, benzene remains both a foundation and a frontier. From a single ring to an entire universe of fused aromatic carbon, its legacy illustrates how fundamental insights can transcend their origins and shape the future of science. In this sense, benzene is not only a milestone of the past, but also a continuing source of inspiration for the chemists of tomorrow.

The Guest Editors gratefully acknowledge the Royal Society of Chemistry for its vision and generous support in bringing this themed collection to fruition, and for fostering a collaborative platform across its journals to celebrate the bicentenary of benzene. We extend our sincere thanks to all contributing authors for their outstanding work, creativity, and commitment, which collectively reflect the structural and functional beauty, vitality and diversity of this field. Finally, we acknowledge the broader scientific community whose continued exploration of aromatic systems ensures that the legacy of benzene remains a dynamic and evolving source of inspiration.

## References

[cit1] Faraday M. (1825). Philos. Trans. R. Soc. Lond..

[cit2] Kekulé A. (1865). Bull. Soc. Chim. Fr..

[cit3] Pauling L. , The Nature of the Chemical Bond, Cornell University Press, 1939

[cit4] Clar E. , Polycyclic Hydrocarbons, Academic Press, London, 1964

[cit5] Kroto H. W., Heath J. R., O’Brien S. C., Curl R. F., Smalley R. E. (1985). Nature.

[cit6] Novoselov K. S., Geim A. K. (2004). et al.. Science.

[cit7] International Agency for Research on Cancer. Benzene , IARC Monographs on the Evaluation of Carcinogenic Risks to Humans, IARC, Lyon, 2018, vol. 120

